# Voting Behavior, Coalitions and Government Strength through a Complex Network Analysis

**DOI:** 10.1371/journal.pone.0116046

**Published:** 2014-12-30

**Authors:** Carlo Dal Maso, Gabriele Pompa, Michelangelo Puliga, Gianni Riotta, Alessandro Chessa

**Affiliations:** 1 IMT, Institute for Advanced Studies, Lucca, Italy; 2 Linkalab, Complex Systems Computational Laboratory, Cagliari, Italy; 3 Department of French and Italian, Pirelli Chair, Princeton Univerity, Princeton, New Jersey, United States of America; University of Maribor, Slovenia

## Abstract

We analyze the network of relations between parliament members according to their voting behavior. In particular, we examine the emergent community structure with respect to political coalitions and government alliances. We rely on tools developed in the Complex Network literature to explore the core of these communities and use their topological features to develop new metrics for party polarization, internal coalition cohesiveness and government strength. As a case study, we focus on the Chamber of Deputies of the Italian Parliament, for which we are able to characterize the heterogeneity of the ruling coalition as well as parties specific contributions to the stability of the government over time. We find sharp contrast in the political debate which surprisingly does not imply a relevant structure based on established parties. We take a closer look to changes in the community structure after parties split up and their effect on the position of single deputies within communities. Finally, we introduce a way to track the stability of the government coalition over time that is able to discern the contribution of each member along with the impact of its possible defection. While our case study relies on the Italian parliament, whose relevance has come into the international spotlight in the present economic downturn, the methods developed here are entirely general and can therefore be applied to a multitude of other scenarios.

## Introduction

A great deal of recent research has been devoted to explaining political polarization in parliaments [1], [2]. This literature has been dominated by models where party polarization is either explained by the polarization of the electorate or through the party and ideology of deputies. A new stem of literature has recently adopted tools of Complex Network Science [3], [4] to investigate this issue, with a network representation being given to committees and subcommittees who share the same members in the US Congress [5], to members of the Congress who co-sponsor bills [6], [7] or those who place the same roll-call votes [8]. We follow the latter approach so that deputies are represented as nodes within a network where the number of shared roll-call votes determines the strength of their links. Similarly to [9], [10] we make use of the network science concept of modularity in order to reconstruct the community structure of the parliament [11]. We introduce a novel method to characterize the position of each deputy in the community of reference, based on its contribution to the modularity score, proposing a more intuitive interpretation compared to that based on the spectral decomposition developed in [10] and in [5]. The method presented here can be easily generalized on a wider European scale, and replicated across a longer time span or in industry-specific policies. In particular, the analysis can be extended to deal with multiple interdependent networks [12] thanks to the interplay between senate and house of representatives or between national and european parliaments and take advantage of recent development in different fields of complex science ranging from critical infrastructures [13], [14] to epidemics transmission [15], [16]. Indeed, nowadays political life of european countries is increasingly connected to, and interconnected through, the European Parliament decisions. Moreover, European parliamentary acts and documents are semantically classified and organized in a EUROVOC thesaurus (http://eurovoc.europa.eu/drupal/), that will make it possible to analyze political positions across different and controlled thematic areas. The rest of the paper is organized as follows. In the “Methods” section we present the methodology used to investigate parliamentary polarization, party cohesion, community structure and their time evolution, in the “Results" section the main findings related to the specific case of the Italian Parliament are presented, while in the “Discussion” section we draw our conclusions and sketch the lines of future research.

## Methods

As the first step in our methodology we construct a graph where each node represents one of the 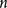 deputies and edges are drawn every time two deputies display the same voting behavior (i.e. both vote in favor, against or abstain from vote. No edges are drawn for absent deputies). We then normalize edges by the total number of votes in the reference period in order to obtain a weighted graph where weights are 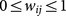. Full weight is given to two deputies 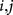 if they participated in all sessions and voted exactly the same way in all of sessions. When a deputy quits the parliament, because of incompatibility, resignation etc., and his or her seat is taken by a new person, we consider the two deputies as being just one node (we check whether this transition leads to some votes in which none of the two deputies had their chairs without finding any discontinuity).

Initially, we look at the topological structure of parties in order to study their cohesion over time. Completely ignoring any *a priori* knowledge of party affiliation, we look at the communities arising directly from voting behavior to see whether they match or not.

### Analysis of party cohesion

Consider each party as a subgraph 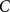 of the graph 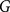, with 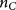 being the number of deputies in the party. An intuitive way of measuring party cohesion (i.e. the tendency of the party to vote as a single entity) is to evaluate the intra-cluster density 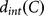 defined as the ratio between the total internal strength of the sub-graph 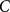 and the number of all possible edges inside that cluster [17]

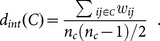



Similarly, we can define the inter-cluster density 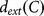 as the ratio between the observed strength of edges running from the nodes of 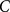 to the rest of the network and the maximum number of edges connecting internal with external nodes: 
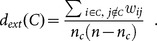



A party stands out as a specific political group if 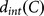 is appreciably larger than the average link density 
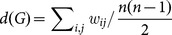
 of the entire network 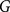 and similarly we expect 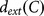 to be appreciably smaller.

Searching for the best tradeoff between a large intra-cluster density and a small inter-cluster one is indeed an implicit or explicit goal for most algorithms used in community detection [11], [17].

### Community and core detection

Modularity optimization is a well-established method for detecting communities [11]. The idea behind modularity is that a random graph should not have a cluster structure so that communities are revealed maximizing the difference between the density of edges in a sub-graph and that expected if edges were connected at random. Hence the modularity function of a weighted graph [18], where in our case nodes are deputies and edges represent the percentage of votes that two of them have in common, is given by: 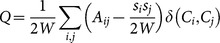



where 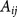 gives the fraction of similar votes deputies 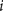 and 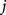 share in common (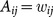), 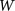 is the total weight in the network, 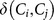 is a delta function that yields one if deputies 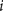 and 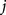 are in the same community (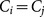) and 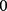 otherwise, and 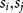 represent the strength of node 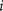 and 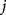 respectively.

In the general case of modern democracies the typical result of the modularity optimization should be the splitting of the graph into two communities that reproduces the government coalition and the opposition.

Moreover each node in its community usually doesn't have the same importance for its stability. Indeed, the removal of a node in the community core should affect the partition much more than the deletion of a boundary node. In other terms, some deputies display such a high degree of internal connections so that they can be identified as the bulk of the coalition. As we proceed toward the boundary, deputies display increasing connections to the opposite community.

In order to investigate this structure, we exploit the properties of the modularity function following a new procedure introduced in [19]. By definition, if the modularity associated with a network has been optimized, every perturbation of the partition leads to a negative variation of the modularity 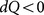.

We compute the effect on the modularity associated with the shift of a deputy from one community to another and we plot the corresponding 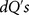 distribution in order to check the coreness of each deputy and his party. In case of three or more communities 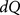 was originally developed in [19] to report the minimum variation in modularity, i.e. modularity was compared against a setup in which each node was moved, one per time, to its *closest* community. Here we rather consider movements to the *farthest* community in order to avoid abrupt shifts in the distribution of 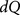 due to the rise of small temporary (third) communities. Finally the histogram of the 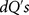 will highlight the different groups that make up the coalition and will show different sub distributions along the support interval of 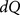.

### Measures of Polarization, Cohesion and Stability

Dealing with roll-call vote's networks as a whole, standard approaches [8], [10], [20] have adopted the modularity score as a measure for party polarization. However, our methodology gives us the possibility to consider the overall voting behavior on a much finer scale, considering the contribution of every single deputy. In line with this, we have decided to measure the polarization as an average decrease in the modularity score consequent to the substitution of two opposite deputies; the larger the decrease in the modularity score, the larger the current contraposition between the two coalitions becomes. So we define the polarization as the sum of the median of the monthly 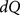 distributions of the two communities (the median has been preferred to the mean as a measure of location, because the distributions of both communities are strongly negatively skewed).

When we focus on features of only one community, we still need to account for the community structure of the whole graph. Think for instance of two time frames in which the members of the ruling coalition vote exactly the same while the opposition voted 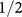 and 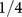 of the time with the government. Then the government 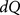 distribution would present more extreme values in the latter case, determining a shift towards more negative values of the mass of the entire distribution, despite the cohesion of the government *per se* not changing at all. Therefore any measure of cohesion should be robust to changes in the location of the distribution. A suitable one is represented by the interquartile difference of the 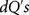 distribution that we will employ as our standard definition for the party/coalition cohesiveness.

In addition to polarization and cohesion, the stability of the government is directly affected by the number of its loyal deputies; in order for laws to be passed, half plus one of the total number of deputies are needed in the Chamber of Deputies. So as a rough rule of thumb, we can consider a government that keeps up to half plus one deputies on his side to still be *safe*. This measure accounts for the stability of the government comunity in the shape of a safety zone that divides the last critical deputy able to break down the majority from the 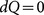 postion before the oppositon community region.

## Results

As a concrete case we analyze the network of deputies in the newly elected Italian Chamber of Deputies (2013). We collect information on the 630 deputies and their voting behavior from the government open data SPARQL endpoint (http://dati.camera.it/sparql). The reader may refer to table 1 for an outline of the main Italian parties mentioned in this paper. The available data cover parliamentary votes from April 2013, when the new parliament was appointed, to the end of December 2013. Despite being quite a short period of time, the dataset covers 2820 parliamentary votes, which implies more than 1,5 million individual votes in our time span. Importantly, the Italian government has made semantic data following W3C standards available, which translates into fast and precise data manipulation through computer based queries. We refer to this source of data for the profiles of deputies and the classification of votes, while data on voting behavior of single deputies was taken directly from institutional web sites (http://documenti.camera.it/votazioni/votazionitutte/FormVotazioni.Asp?Legislatura=XVII).

**Table 1:** Outline of the main Italian parties.

Party	Coalition	%*	Notes
Partito Democratico (PD)	Gov	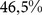	Main center-left party, historically lead by Prodi
Il Popolo della Libertà (PDL)	Gov/Opp	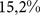	Main conservative party, lead by Berlusconi
Forza Italia (FI)	Opp	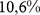	From PDL split, founded and lead by Berlusconi
Nuovo Centro Destra (NCD)	Gov	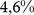	From PDL split, lead by Alfano
Scelta Civica (SC)	Gov	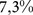	Lead by Monti, PM for one year after 2011 crisis
Movimento 5 Stelle (M5S)	Opp	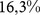	Lead by comedian Grillo, form of *direct* democracy
Sinistra-Ecologia-Libertà (SEL)	Opp	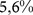	Left party, former ally of PD
Lega Nord (LN)	Opp	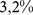	Autonomist party of Northern Italy, former ally of PDL

*Shares updated to may 2014, smaller parties omitted.

**Figure 1:**
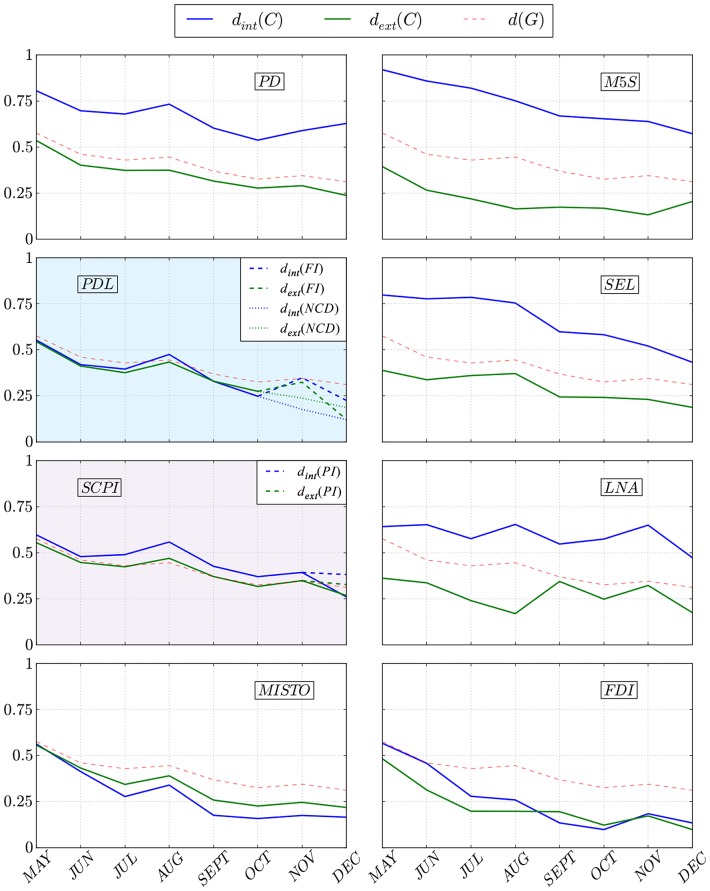
Members of a party show cohesion if the links connecting them are stronger than the ties with other deputies. We capture the former by the intra-cluster density 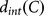 and the latter by the inter-cluster density 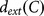. The party shows high cohesion when the two lie considerably higher and lower respectively copared to the average link density of the whole parliament 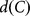.

**Figure 2:**
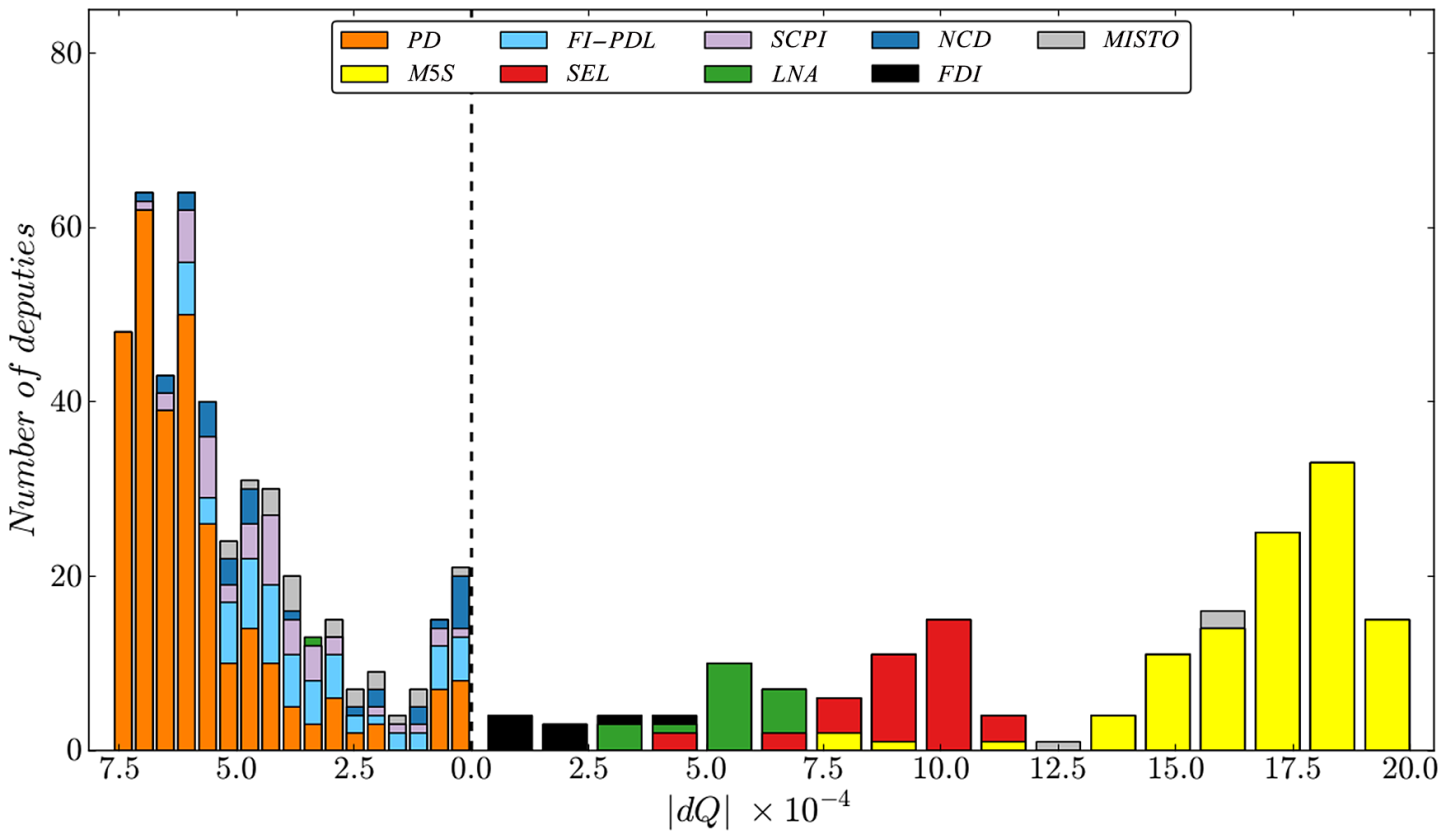
Community Structure of the Italian Parliament. The vertical dashed line separates the two main coalitions/communities (Government/Opposition). Each coalition comprises different parties corresponding to different colors. The quantity ‘dQ’ is associated to the coreness of each deputy/party. The distributions are obtained computing the coreness of each deputy and then aggregating them in the form of a stacked istogram. The more the distance of the bars from the vertical dashed line, the more the deputies/parties are at the core of their coalition. Notice how the main parties tend to segregate in clusterd distributions with different positions in the ‘dQ’ axes.

Fig. 1 represents the evolution of density measures over time for each party in the Italian Chamber of Deputies. While the structures of the M5S, PD, SEL and LNA parties are recognizable within the graph, the other groups present inter- and intra-cluster densities that are very close to each other, or at times even overlapping. This means that at a certain point their votes proportionally coincide to a greater degree with other groups than with their own members. The plots marked with a colored background report the splitting of two political groups, when the PDL breaks up into the NCD and the FI in November 2013 and the PI exits from the SCPI in December 2013. The inter-cluster density, represented in green, is clearly higher for groups who support the government (PD, PDL and SCPI). Theoretically these groups should vote in compliance with the majority's prescriptions, thereby showing a similar voting behavior. Once we take into account the average monthly levels of edge density 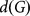 the topological structure of parties becomes very similar to the rest of the graph. As such, parties may not be the most appropriate representation of voting structure, thus leading us to consider the behavioral identification of political groups through the modularity function. Once applied to the graph of deputies, the modularity optimization usually splits the graph into two communities that almost exactly match the government coalition and the opposition as shown in Fig. 2 where the vertical dashed line separates the two coalitions.

Afterword we compute the effect on the modularity associated with the removal of a deputy from his community computing the corresponding 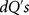 and the result is also shown in Fig. 2.

The histogram shows the 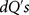 distribution of the government's coalition on the left side of the dashed line and that of the opposition on the right, with alle the dQ associated to different parties in different colors.

**Figure 3:**
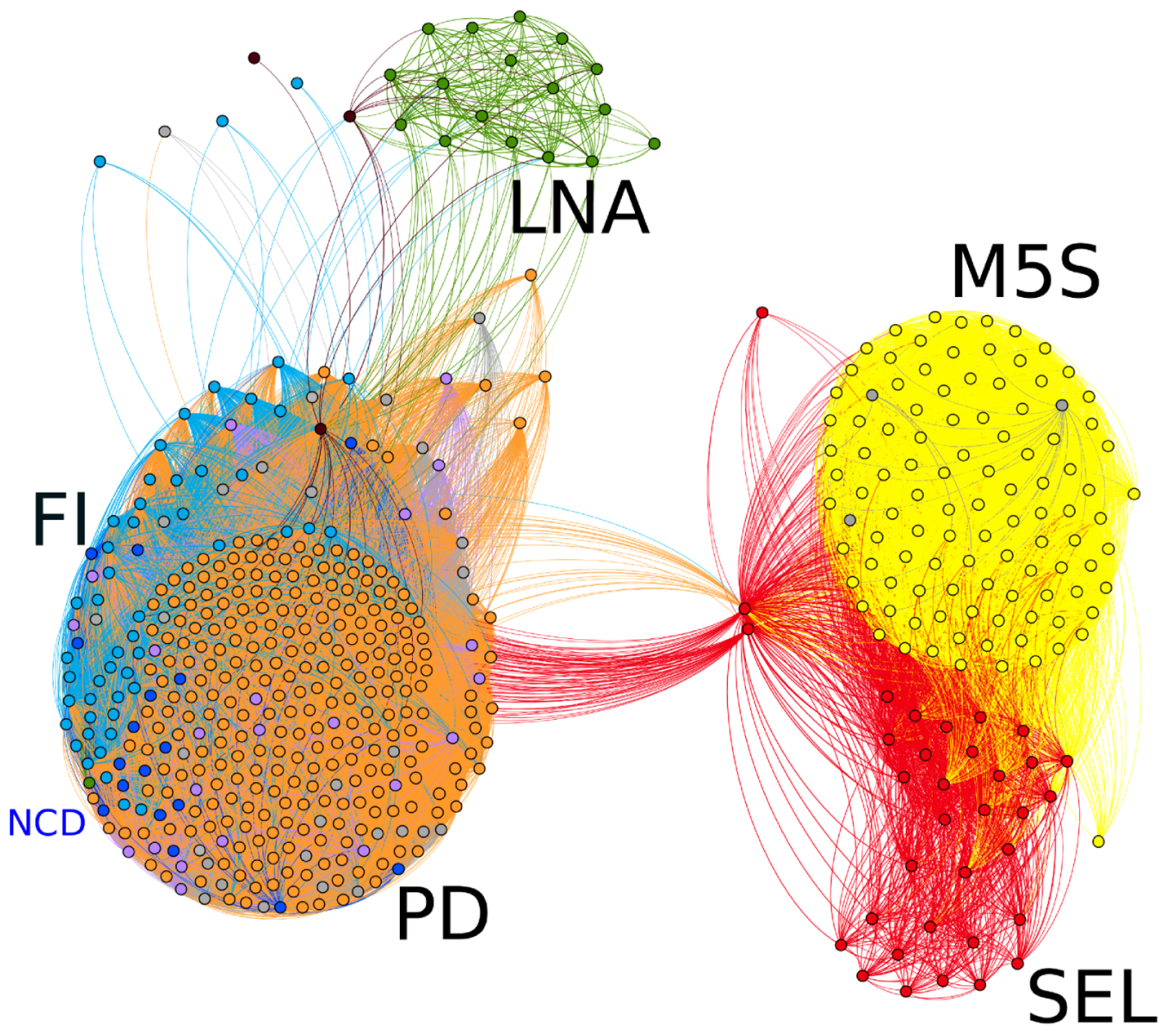
Network Structure of the Italian Parliament. Here we show the arrangement of deputies/nodes in the network of their relationships containing the information of all the votes in the time frame we considered. We highlight with different colors, the different parties.

In order to have a direct view of the actual network structure we show in Fig. 3 the clusterization of the various parties, with different colors of the deputies/nodes. Indeed, the core of the coalition appears to be made up by a relatively higher share of deputies from the center-left party PD while relatively more deputies from the center-right party PDL appear to be at the periphery as we move to the right. This provides an interesting insight on the rather different roles played by the two main Italian parties joined by a coalition pact, namely the PD and the PDL, with the latter ultimately quitting the government in mid November 2013. As for the opposition, note that the support of 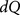 is far more dispersed with each group taking on a limited range of values in the distribution. This is not surprising in that the opposition is not a coalition *per se* but rather a set of groups that might vote with the ruling coalition depending on the subject at hand. In particular, deputies from the M5S make up the core of the opposition with a higher magnitude of 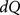, which also holds true when compared to the core of the government coalition. This may be due to a relatively inflexible opposition to the government or in equal measure to the fact that it is the largest group in the opposition community. On the other hand, the SEL and the LNA are progressively closer to the border of the community, which may be reasonable if we consider that these groups used to be allies of two parties in the government coalition, namely the PD and the PDL respectively.

### Time evolution of the community structure

**Figure 4:**
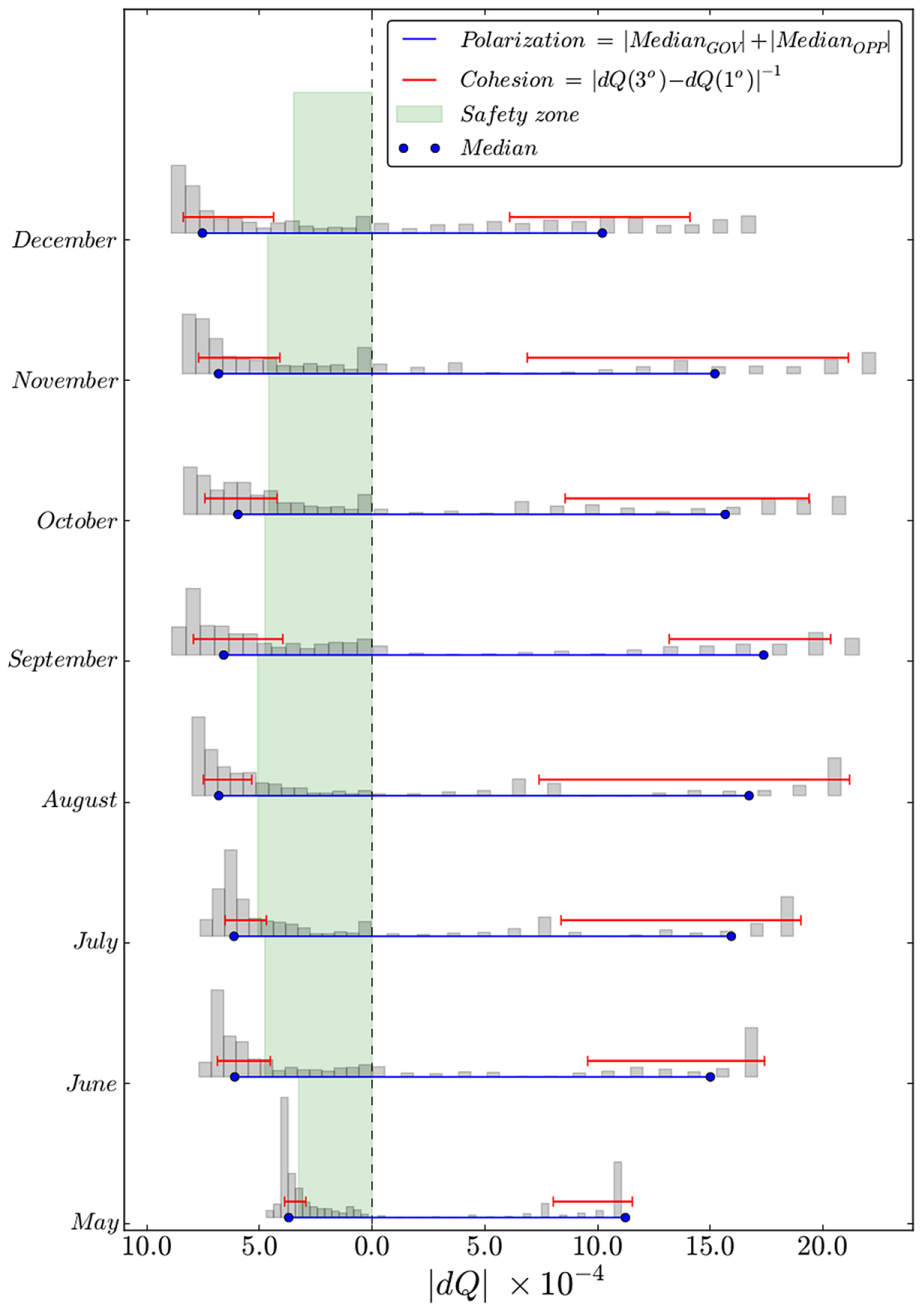
The evolution of community structure over time provides a way to track the cohesion of the government and the overall polarization in the parliament. The empyrical analog of the cohesion is represented here by the interquantile difference of the 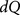 distribution, where higher cohesion occurs for lower values of the interquantile. On the other hand higher parliament polarization is captured by the distance between the two medians. Finally the *safety zone* that divides the last critical deputy able to break down the majority from the vertical dashed line (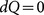) is represented in green.

The same analysis has been carried out over time, dividing votes per month, building up the corresponding graphs and performing the community and core analysis on each monthly network. In Fig. 4 the two main communities present increasingly extreme values of 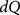 over time, which in turns provides evidence of increasing polarization in the parliament, as it is measured as the sum of the median of the monthly distributions (see Methods section).

This may reflect the change in the political position of M5S which moved from declared openness to the government on a single bill basis [21] to a very sharp contrast as events unfolded. Two controversial bills that occupied a large fraction of the assembly's sessions over summer 2013 (the shut down of an old iron factory and a bill [22] containing economic reforms to tackle the crisis culminated with M5S's deputies blocking the assembly and then leaving it once the measures were eventually passed) and the worsening of the political climate that led to the repeated demand for resignation of government ministers in the following autumn [23], might have driven the political debate towards increasingly polarized configurations as it is evident in Fig. 4 also on a monthly level of aggregation.

In this respect, December noticeably stands out, with a reduction in the extreme values of 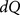 for the opposition. This is actually driven by the fragmentation of the PDL, which witnessed its deputies loyal to the leader Silvio Berlusconi, withdraw their support of the government [24] and start to vote with the opposition to the point of being identified as part of it at least in its border. The Fig. 4 illustrates also the cohesion, or rather its flip side: the heterogeneity of deputies within a single community, along with the government stability represented through a green *safety zone*. This area spans values of 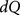 smaller (in absolute value) than the monthly critical value 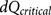. The latter corresponds to the level of coreness of the deputy which would pose the government in numeric inferiority, were he leaving the coalition. In the specific case of the Italian Parliament, the Chamber of Deputies has 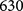 representatives and the critical value will correspond to the 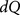 relative to the 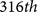 deputy.

**Figure 5:**
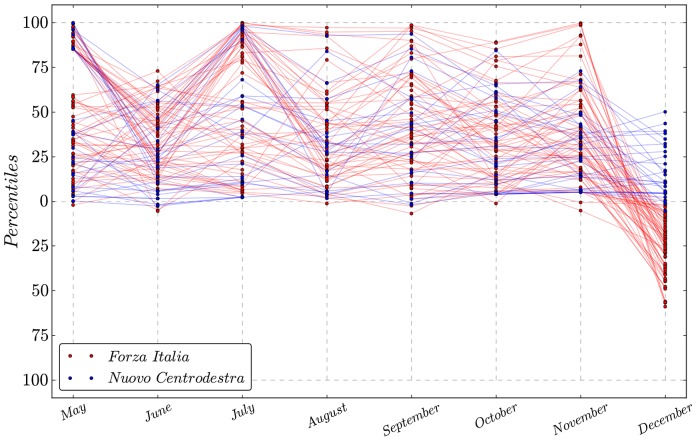
The position of single deputies within communities provides insights on what happens when a party splits up. In this particular case the PDL party in mid November 2013 splits into two different parties ‘Forza Italia’ and ‘Nuovo Centrodestra’. Interestigly, nodes at the core of the government coalition become core in the opposition one when the split up occurs. This is evidence of political voting being driven by coalitions' affilitions rather than the policy content of each roll call vote.

With fixed levels of polarization and cohesion, a greater absolute value of 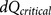 would widen the *safety zone* in that a relatively more loyal deputy would have to leave the government coalition in order to make it facing the risk of having its laws rejected. Having investigated the peculiar structure of the government coalition, we focus on a political party that may be partly responsible for the variability of the coalitions topology over time. Indeed the PDL, after a long debate regarding whether to support the government or not, eventually split into two different parties. After the split in mid November, deputies from the FI moved into the opposition community. However, surprisingly, those who left moved from the core of the government to relatively core positions in the opposition, as reported in Fig. 5.

This dynamic may somehow explain the peculiar drop of the polarization observed in December in Fig. 4, as the FI group switched voting behavior to such a degree as to be recognized as part of the opposition, simultaneously reducing the contraposition between the two communities.

## Discussion

The study of the consensus dynamics in modern parliamentary democracies is of great importance for the validation by citizens of the performance of their representatives. These dynamics are often hidden by complicated voting procedures that prevent the easy identification of these civil representatives. We need new ways to look at the details of the political activities, which go beyond the standard statistical indicators, ways that are able to reveal the dynamics of the general organization of the government, its opposition and even their internal structures, in a format that is intelligible to non-expert users. In this study we introduced a novel procedure to map parliamentary voting trends onto a network structure in which the nodes are the deputies and the edge weights are the strength of their relations. These weights, month by month, quantitatively measure the degree of closeness between couples of deputies as the number of votes they shared in a specific time frame. Once this network has been built up, using Community Detection techniques borrowed from Complex Network Science, it is possible to reconstruct the main coalitions, the government and the opposition from the bottom up; through a ‘Core Detection' analysis it is also possible to uncover the internal structure of these aggregations. Using the leverage of later analyses we were able to quantitatively detect the position of each party, the strength and consistency in its coalition and the level of polarization between government and opposition.

Furthermore, the Open Data movements around the world are pushing public administrations to provide free and open access to massive amounts of data, which can be used by citizens and companies as a starting point for the detailed analysis of public policies. In this study, we relied on a recent service introduced by the Italian parliament that allows the automated extraction of certified information about the votes of the Chamber of Deputies. Through this service we have been able to perform a thorough analysis of the dynamics of the Italian parliamentary factions over nearly a year of legislation, using the aforementioned methodology.

These methods open up new possibilities of bringing citizens closer to their representatives, thereby establishing the foundations for a more transparent democracy.
